# Timely Palliative Care Could Be Another Benefit for Cancer Patients with Non-Malignant Pain

**DOI:** 10.3390/cancers15092588

**Published:** 2023-05-02

**Authors:** Marco C. Maltoni, Costanza M. Donati, Romina Rossi, Alessio G. Morganti

**Affiliations:** 1 Oncology Unit, IRCCS Azienda Ospedaliero-Universitaria di Bologna, 40138 Bologna, Italy; 2 Medical Oncology Unit, Department of Specialized, Experimental and Diagnostic Medicine (DIMES), Alma Mater Studiorum-University of Bologna, 40126 Bologna, Italy; 3 Radiation Oncology, IRCCS Azienda Ospedaliero-Universitaria di Bologna, 40138 Bologna, Italy; 4 Radiation Oncology, Department of Medical and Surgical Sciences (DIMEC), Alma Mater Studiorum-University of Bologna, Via Albertoni 15, 40138 Bologna, Italy; 5 IRCCS Istituto Romagnolo per lo Studio dei Tumori (IRST) “Dino Amadori”, 47014 Meldola, Italy

**Keywords:** timely palliative care, non-malignant pain, pain management, editorial

Cancer patients, as well as individuals in the general population, suffer from non-malignant pain (NMP), although with variable prevalence in the few studies dealing with this topic [1,2,3,4,5]. Furthermore, there are no national or international guidelines concerning this peculiar condition. However, two papers recently published in *Cancers* [6,7] have addressed this issue, even if they reported completely opposite results.

In the first study, Hui et al. presented the results of a prospective cross-sectional survey of 200 cancer patients referred to a supportive care clinic [6]. In their analysis, the authors observed that approximately one-third of patients (67) had NMP and that 49% of patients with NMP alone were treated with opioid medications. The authors noted that, despite the fact that the use of opioids in NMP is to be avoided or at least minimized, the recorded rate of patients treated with these drugs is clearly excessive. Therefore, they concluded by underlining the need to develop specific guidelines for NMP management in cancer patients [6].

Unlike the previous analysis, a prospective observational study conducted by our group involved 2104 cancer patients evaluated and treated in 15 radiotherapy centers [7]. Within this setting, the adequacy of the analgesic drug therapy was assessed using the Pain Management Index as a tool. Overall, the study showed that the prevalence of patients with inadequately treated pain is 45.0%. Furthermore, the rate of patients with poorly managed pain was 30.5% in cancer pain and 71.5% in NMP (*p* < 0.001). Specifically, none of the patients with mild NMP (Numeric Rating Scale, NRS: 1–4) were taking analgesics. Additionally, of patients with moderate NMP (NRS: 5–6), 38.3% were not taking analgesics, and 61.7% were taking non-opioid medications. Finally, of patients with severe NMP (NRS: 7–10), 33.3% were not taking analgesics, 54.5% were taking non-opioid analgesics, and only 12.2% were taking opioid medications.

The authors concluded that the management of NMP is poorly adequate in the oncological setting. Therefore, educational strategies on pain management for radiation oncologists and a multidisciplinary management of this symptom are necessary to improve patients’ quality of life. More generally, attention should be paid to the treatment of NMP in cancer patients, considering that this condition seems largely neglected [7].

The clear differences in the results of these two studies can be explained by the different settings of the analyzed patients. Indeed, Hui et al. enrolled patients referred to palliative care and, therefore, in a setting with doctors probably more familiar with the use of stronger drugs, even in subjects with NMP [6]. In contrast, the study by Donati et al. was conducted on patients referred to radiotherapy centers, and only 54.2% and 52.0% of them had advanced/metastatic disease or were referred for palliative treatment, respectively [7]. Furthermore, the study by Hui et al. was conducted in the USA [6], while the one by Donati et al. was performed in Italian radiotherapy centers [7]. Geographical differences in the adequacy of pain therapy, even within the same country, were previously reported by Donati et al. [7] and by Shen et al. in a Taiwanese study [8].

Furthermore, it is possible that the different outcomes of these two studies result from two different attitudes in the management of NMP. In particular, the excessive use of opioids recorded in the analysis by Hui et al. could arise from the pragmatic consideration that, given the poor prognosis of patients in palliative care, the risk of long-term misuse or abuse of opioids is very small [5]. On the contrary, the undertreatment of NMP recorded in our study [7] could be explained by greater attention, in cancer patients, only to tumor-related symptoms.

Ultimately, it is interesting to note how, albeit in opposite directions, these two studies [6,7] show a clear inadequacy in the management of NMP in cancer patients. 

It is also interesting to consider these results in light of a recent literature review, also published in *Cancers* and authored by the team of Hui et al. [9], about the concept of “timely palliative care” (TPC). This term indicates an optimization and personalization of the referral processes to palliative centers by delivering supportive therapy in suitable settings and with optimal timing. More specifically, TPC is an evolution of “early palliative care” aimed at reducing the problems of the poor feasibility of the latter due to economic and organizational constraints. In fact, where early palliative care generally consists of the systematic referral of patients to palliative therapies within 2–3 months from the diagnosis of advanced cancer, in any case when the ECOG performance status is still ≤2, the start of TPC depends on the real patient needs.

The authors of the review emphasized that requirements for an effective implementation of TPC are as follows: (i) systematic screening of the need for supportive care in patients in oncology clinics, (ii) the shared definition of institution-specific criteria for the activation of assistance, and (iii) the availability of the necessary resources. Furthermore, the authors pointed out that the advantages of TPC are multiple. Among these, the authors highlighted: (i) the proactive prevention of symptoms with reduced access to emergency rooms and hospital admissions, (ii) a greater number of visits to the patients, and therefore a more effective management of symptoms and psychological support, and (iii) adequate time to establish discussions with the patient on sensitive topics, such as the communication of the prognosis and end-of-life decisions [9].

Therefore, considering the studies of Donati et al. [7] and Hui et al. [6], it can be argued that an improved management of NMP in cancer patients can be added to the clear benefits of TPC (Figure 1). In fact, NMP is a complex problem being the few available studies retrospective and not focused on NMP as a primary objective (Table 1) [1,2,3,4,5,6,7,8]. Furthermore, specific guidelines for the management of NMP in cancer patients are lacking. Indeed, guidelines on NMP explicitly exclude cancer patients, while guidelines on pain management in cancer patients do not include the treatment of NMP. In reality, as highlighted by Hui et al., the management of NMP in cancer patients presents very specific and complex problems [6].

Other studies are needed to further clarify these observations and, in particular, to identify patients with the highest risk of inadequately treated NMP and to evaluate the real impact of TPC in avoiding poor NMP management.

In conclusion, to the known advantages of TPC and pending consensus guidelines on NMP in the oncological field, we should add the possibility of optimizing pain management in this particular setting.

## Figures and Tables

**Figure 1:**
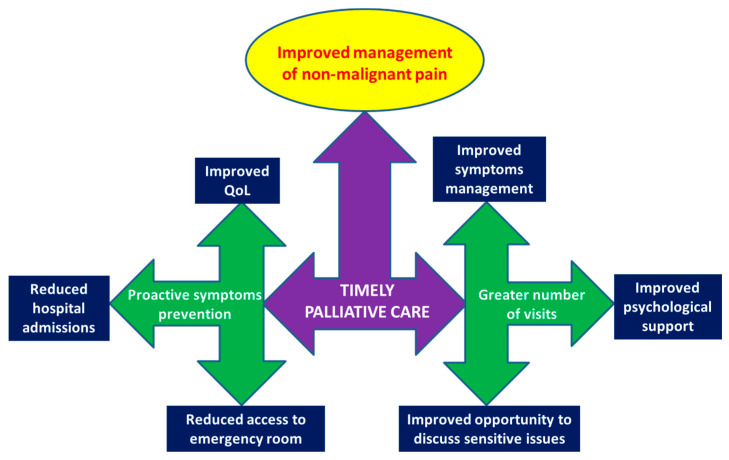
Improved management of non-malignant pain in cancer patients could be an additional advantage of timely palliative care.

**Table 1:** Characteristics and results of studies on non-malignant pain in cancer patients.

Author	Center	No. of Patients (Patients with Pain/Total)	Setting and Methods	Results
Stromgren A.S., 2004 [1]	Department of Palliative Medicine, Bispebjerg Hospital, Copenhagen, Denmark	144/175	Prospective analysis of pain characteristics and treatment outcome of specialized palliative care	NCP: 4.9%
Massaccesi M., 2013 [2]	Università Cattolica del S. Cuore, Campobasso, Italy	398/865	Prospective analysis of PMI during the initial assessments or follow-up visits of cancer patients in a radiation oncology unit	PMI < 0: 82.6%; PMI (only NCP): 91.4%
Barbera L., 2013 [3]	Odette Cancer Centre, University of Toronto, Toronto, Ontario, Canada	9826/9826	Prospective analysis of opioid prescriptions for elderly cancer patients reporting pain	NCP: 15.0% untreated NCP: 40%
Childers J.W., 2015 [4]	Department of Medicine, Division of General Internal Medicine, Section of Palliative Care and Medical Ethics, University of Pittsburgh, Pittsburgh, PA, USA	323/323	Retrospective chart review of patients evaluated in a cancer pain and supportive care clinic	NCP: 26.6%
Gauthier L.R., 2009 [5]	Department of Medical Oncology, Princess Margaret Hospital, University Health Network, Toronto, Canada	81/81	Prospective analysis of pain acceptance in patients attending the Palliative Care, Pain, Gastrointestinal, and Lung clinics	NCP: 43.2%
Hui D., 2020 [6]	Department of Palliative Care, Rehabilitation and Integrative Medicine, MD Anderson Cancer Center, Houston	200/200	Prospective cross-sectional survey of cancer patients referred to a palliative care clinic	NCP: 33.5% (on opioids: 49%)
Donati C.M., 2022 [7]	Radiation Oncology, Bologna University, Bologna, Italy	1409/2104	Observational prospective analysis of PMI in patients treated in 13 radiation oncology departments	NCP: 32.4% PMI < 0 (NCP): 71.5%; PMI < 0 (total): 45.5% NCP (NRS: 7–10): 12.2% on opioids
Shen W.C., 2017 [8]	Division of Hematology-Oncology, Linkuo Chang Gung Memorial Hospital and Chang Gung University, Taoyuan, Taiwan	1659/2652	Observational prospective analysis of PMI in outpatients treated in 16 centers (oncologic clinics)	PMI < 0: 32.4%; higher PMI < 0 rates are correlated with female gender, breast cancer, NCP, hospitals in northern Taiwan

Legend: PMI: Pain Management Index; NCP: non-cancer pain.
